# Personalised Medicine in Cervical Cancer: Evaluating Therapy Resistance Through Multi‐Model Approaches

**DOI:** 10.1002/cam4.70995

**Published:** 2025-06-26

**Authors:** Madré Meyer, Carla Eksteen, Cayleigh de Sousa, Nireshni Chellan, Ruzayda Van Aarde, Meenal Bhaga, Johann Riedemann, Matthys H. Botha, Frederick H. van der Merwe, Anna‐Mart Engelbrecht

**Affiliations:** ^1^ Department of Physiological Sciences Stellenbosch University Cape Town South Africa; ^2^ South African Medical Research Council (SAMRC) Cape Town South Africa; ^3^ CancerCare, Cape Gate Oncology Centre Cape Town South Africa; ^4^ Department of Obstetrics and Gynecology Stellenbosch University Stellenbosch South Africa

**Keywords:** cervical cancer, personalised medicine, senescence, treatment resistance

## Abstract

**Introduction:**

Cervical cancer remains a leading cause of malignancy among women globally, disproportionately affecting women from low‐to‐middle‐income countries, including South Africa. The high prevalence in impoverished communities places significant pressure on the public healthcare system. In these regions, human papillomavirus (HPV); the primary risk factor for cervical cancer—along with co‐occurring immunosuppressive conditions such as HIV, is common. Compounding this burden is the widespread development of treatment resistance to conventional therapies like cisplatin and carboplatin. Resistance is frequently associated with therapy‐induced cellular senescence, underscoring the need for more personalised treatment strategies tailored to individual patient profiles.

**Methods and Materials:**

This study aimed to assess ex vivo methods' utility in predicting patient‐specific therapy responses. Biopsy samples from cervical cancer patients were cultured and subjected to various chemotherapies. Cell viability, senescence markers and treatment resistance pathways were analysed to determine optimal treatment outcomes.

**Results:**

The findings revealed significant variability in optimal treatment responses, with ex vivo methods demonstrating limitations in fully capturing the complexity of patient‐specific reactions to therapy. No single experimental model provided comprehensive predictive insights into treatment outcomes.

**Conclusion:**

This study underscores the need for integrative and multidisciplinary approaches when evaluating treatment strategies for cervical cancer. While ex vivo models offer valuable insights, combining multiple experimental methods is crucial for a more reliable and comprehensive understanding of treatment response and resistance mechanisms. Standardiszing approaches or employing method combinations may enhance personalised medicine efforts, particularly in resource‐limited settings.

## 1 Introduction

Cervical cancer ranks as the fourth most prevalent malignancy among women worldwide [1], with a disproportionate impact on low‐to‐middle‐income countries (LMICs) like South Africa [2]. It is the leading cause of cancer‐related death among women in Sub‐Saharan Africa, primarily due to the high prevalence of Human Papillomavirus (HPV) infection, particularly oncogenic subtypes HPV‐16 and HPV‐18 [3]. Despite advancements in screening and HPV vaccination, cervical cancer remains a major health threat. A key factor contributing to treatment resistance is oncogene‐induced senescence, a hallmark of cancer, defined as a state of irreversible growth arrest triggered by endogenous and exogenous stressors. Senescent cells can accumulate in cervical tissues, creating a tumour‐promoting microenvironment via the activation of pathways such as p16/Rb and secretion of senescence‐associated secretory phenotype (SASP) proteins [4, 5]. This senescent phenotype is linked to chemotherapy resistance and cancer recurrence via multidrug resistance mutation 1 (MDR1) and ATP binding cassette subfamily G member 2 (ABCG2) [6]. However, the role of senescence‐induced treatment resistance in cervical cancer remains poorly understood, underscoring the need for innovative approaches, such as personalised medicine, to enhance our understanding thereof. We hypothesised that patient‐derived biopsies could facilitate personalised treatment predictions. This study aimed to investigate this association using an ex vivo approach to assess individual cervical cancer patients' chemotherapy responses, emphasising the importance of multidisciplinary methodologies in personalised medicine.

## 2 Methods and Materials

### 2.1 Patient Recruitment

Ethical approval was granted by Stellenbosch University (HREC—N22/04/037). Participants were recruited from Tygerberg Hospital's Obstetrics and Gynaecology outpatient clinic, where they provided informed consent. Inclusion criteria were: (1) advanced cervical cancer diagnosis, (2) female at birth, (3) fluent in English, Afrikaans or isiXhosa, (4) 18 years or older, (5) no history of other cancers and (6) treatment‐naïve status. Patients were not excluded based on socioeconomic background, education level or race. Identities were anonymised using unique identifiers.

### 2.2 Collection of Specimens

During routine staging (based on the International Federation of Gynaecology and Obstetrics [FIGO] system) small cervical tumour biopsies (±1 to 2 g) were collected, placed in Dulbecco's Modified Eagle Medium (DMEM), and kept on ice. Samples were processed at Stellenbosch University's Department of Physiological Sciences, mechanically dissociated, and cultured until confluency before being seeded into culture flasks and treated with low‐dose chemotherapy. A retrospective analysis of patient data was performed to explore correlations between senescence markers and treatment responses.

### 2.3 Primary Cell Culture

**FIGURE 1:**
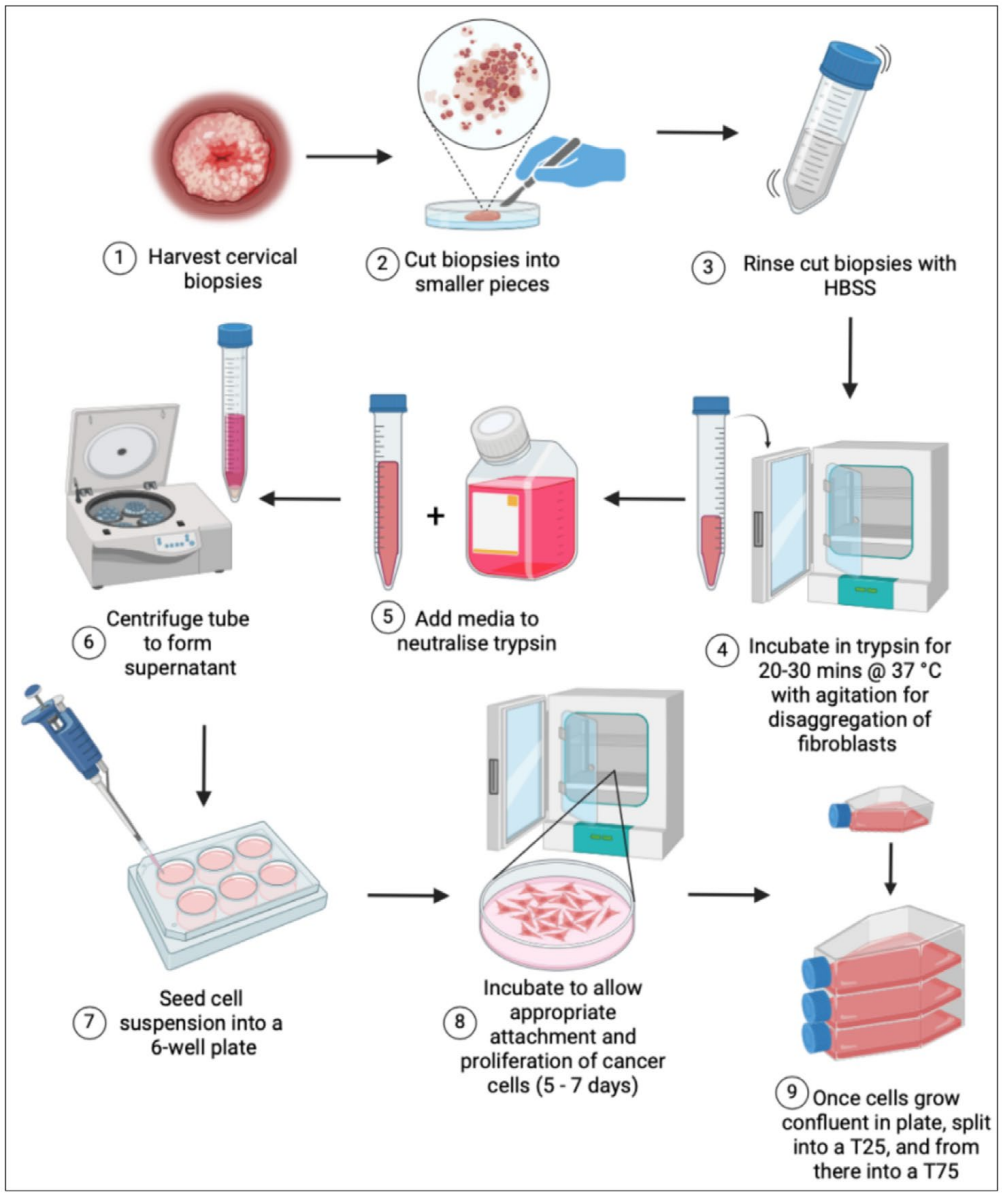
Protocol for the dissociation and processing of primary cervical tumour biopsies (made with BioRender). HBSS, Hank's buffer saline solution.

As seen in Figure 1, tumour biopsies were mechanically dissociated, incubated with 0.25% Trypsin–EDTA, centrifuged at 1500 rpm for 3 min, and seeded into 6‐well plates containing DMEM/F12 (50:50) supplemented with 10% fetal bovine serum, 1% Penicillin–Streptomycin and 1% Amphotericin (Gibco, ThermoFisher Scientific). Cells were cultured at 37°C with 5% CO_2_ and monitored for contamination and successful attachment. Once 75% confluency was reached, cells were trypsinised and reseeded.

Primary cell cultures offer a more accurate representation of the tumour microenvironment but pose challenges such as slow proliferation, poor adherence, fibroblast overgrowth, contamination risks and limited passage numbers [7]. Despite optimisation attempts, only 50%–60% of patient samples reached a viable stage for experimentation, complicating efforts to determine optimal treatment responses. These challenges are further discussed in subsequent sections.

### 2.4 Treatment Protocol

Six treatment groups were used, along with the patient controls: (1) 0.005 μM Paclitaxel (Pxt), (2) 1 μM Doxorubicin (Dxr), (3) 3.3 μM Gemcitabine (Gem), (4) 12 μM Carboplatin (Carbo), (5) 12 μM Carbo +0.005 μM Pxt and (6) 12 μM Carbo +3.3 μM Gem. Low‐dose chemotherapy levels were selected based on the literature [8, 9, 10].

### 2.5 WST‐1 Cell Viability Assay

Cell metabolic activity was assessed using the WST‐1 assay (Merck, Sigma‐Aldrich). The reagent was added (1:10 ratio) and incubated for 4 h, after which absorbance was measured at 450 nm using an EL 800 Universal Microplate Reader.

### 2.6 Western Blots

Protein extraction was performed 72 h post‐treatment. Cells were washed with PBS, lysed in modified RIPA buffer and centrifuged at 4°C. Protein quantification was done using the Bradford Assay. Samples were prepared in Laemmli buffer and run on 12% resolving and 4% stacking gels (Bio‐Rad TGX Stain‐Free FastCast Kit). Membranes were blocked in 5% Bio‐Rad Blocking Buffer, incubated with primary antibodies (MCM2, p16, ABCG2, MDR1, p38, Rb, p‐Rb; Cell Signalling Technology), and treated with secondary antibodies (1:10,000 dilution). Chemiluminescent signals were detected using Image Lab software and normalised to GAPDH.

### 2.7 Senescent Phenotype Determination via Senescence‐Associated Beta‐Galactosidase (SA‐ß‐Gal) Staining Kit

SA‐β‐gal staining (Cell Signalling Technology) was performed 72 h post‐treatment to assess senescence. This histochemical assay detects β‐galactosidase, a lysosomal enzyme expressed in senescent cells at pH 6 [11]. Cervical cells (10,000 per well) were seeded in 12‐well plates, treated, fixed and stained per the manufacturer's protocol, and then incubated overnight without CO_2_. Staining was visualised using a Zeiss phase‐contrast microscope and analysed with ImageJ (FIJI).

### 2.8 Blood Plasma Inflammatory Profiling

Inflammatory cytokines (IL‐1β, IL‐6, TNF‐α, VEGF‐α, IFN) were quantified using the Milliplex MAP Human Cytokine/Chemokine Magnetic Bead Panel (Merck). Plasma samples were thawed from −80°C to −20°C. Beads bound to analytes were incubated with biotinylated antibodies and Streptavidin‐PE, then analysed via Luminex flow cytometry.

### 2.9 Statistical Analysis

Visual representation and statistical analysis of the data were generated using GraphPad Prism version 9 for Mac OS X (GraphPad Software, San Diego, CA). All the values represented were expressed as a percentage of the control ± standard error of the means (SEM). Significant differences were assessed by one‐way analysis of variance (ANOVA) using the Tukey *post hoc* test. A *p*‐value < 0,05 was considered statistically significant.

## 3 Case Report for the Patient

This case provides valuable insights into the potential of our approach, even within the limitations faced with primary cell cultures, while also highlighting the importance of employing integrative and more advanced methods.

A 32‐year‐old woman, referred to as ‘the patient’, was diagnosed with stage 3B cervical squamous cell carcinoma (SCC) during a staging appointment at Tygerberg Hospital (Cape Town, South Africa). This is unusually young for such an advanced diagnosis [12]. More than 95% of cervical SCC cases are HPV‐positive, a figure even higher in sub‐Saharan Africa. The patient was also HIV‐positive, a co‐infection that increases the likelihood of cervical cancer by up to six times [13]. Following staging and obtaining informed consent, a cervical tumour biopsy was taken and processed for further analysis as described in Section 2.1.

### 3.1 Cell Viability Assay

**FIGURE 2:**
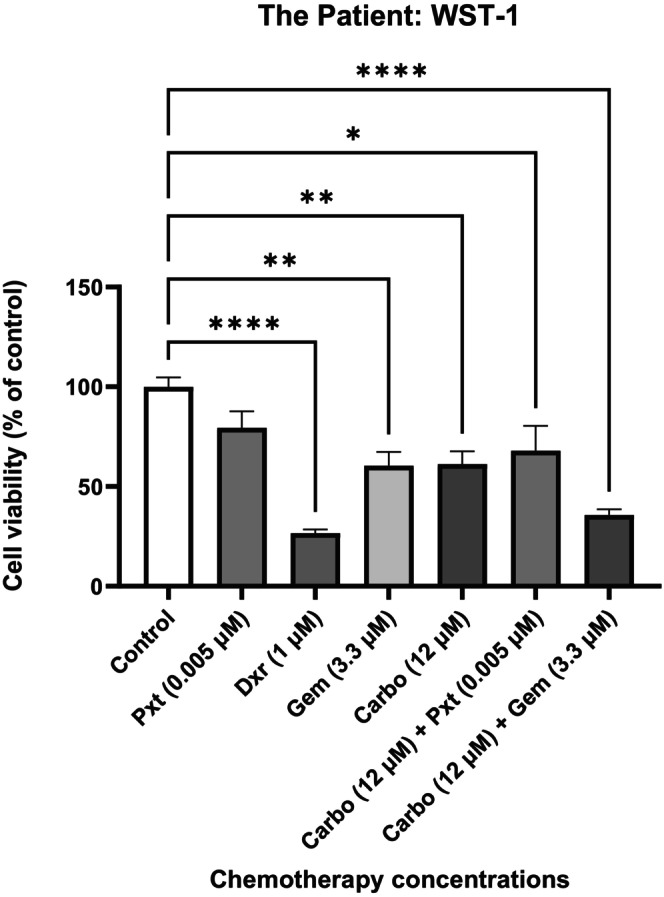
Percentage viability of the patient cells after treatment determined via WST‐1 assay. The patient's cells were seeded at a density of 5000 cells per well and treated for 72 h to allow for appropriate senescence induction. Results are presented as means ± SEM (*n* = 3). The asterisk (*) denotes *p* < 0.05, (**) denotes *p* < 0.01 and (****) denotes *p* < 0.0001. Carbo, carboplatin; Dxr, doxorubicin; Gem, Gemcitabine; Pxt, paclitaxel.

Absorbance results of the cellular viability assay are presented in Figure 2 and are expressed as a percentage of the patient control. There was a significant reduction in cellular viability in all the chemotherapy groups, except for the Pxt treatment group. Additionally, the greatest reduction in cell viability was seen in the Dxr group (*p* < 0,0001) and the Carbo + Gem combination treatment group (*p* < 0,0001).

Dxr treatment exhibited the most significant decrease in cell viability (*p* < 0.0001). Carbo, Pxt, Gem and/or combinations are typically administered for cervical cancer patients. Tygerberg Hospital, where the patient's samples were obtained, exclusively administers platinum‐based chemotherapy, cisplatin, with adjuvant radiation therapy [14]. Nevertheless, there is evidence of studies that investigated the use of Dxr on cervical cancer cell lines; for example, a study by Bano et al. [15] demonstrates that Dxr, combined with Chloroquine, reduces cell viability and increases apoptosis in HeLa cells. However, challenges with Dxr, including poor solubility and severe side effects like myocardial infarctions, often limit its clinical use [16]. In this study, Carbo reduced cell viability by approximately 40% compared to the control (*p* < 0.01), whereas Dxr reduced viability by about 75%. Combination therapies further showed promise. The Carbo + Gem group achieved a greater reduction in viability (~70%, *p* < 0.0001) than Carbo alone, consistent with studies on SiHa and CaSki lines that highlight synergistic interactions between Gem and Carbo [17]. Similar findings have been reported for cisplatin and Gem combinations [18]. Despite these promising results, Gem and Carbo's combinations remain outside South Africa's standard treatment protocols [14].

### 3.2 SA‐ß‐Gal Staining for Confirmation of Senescence Induction

**FIGURE 3:**
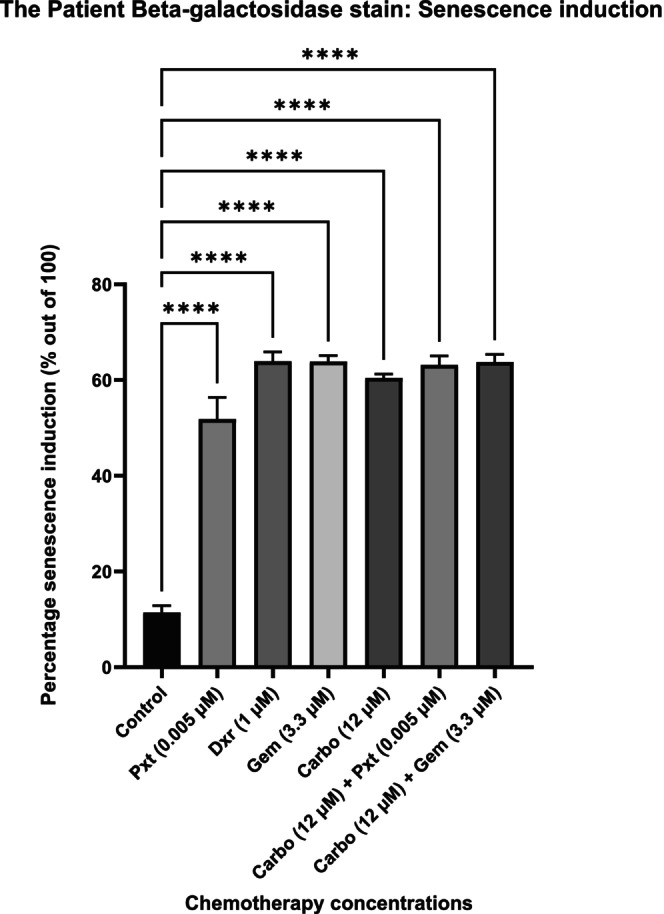
Percentage chemotherapy‐induced senescence induction in the patient cells, determined via beta‐galactosidase staining. Cells were seeded at a density of 10,000 cells per well and treated for 72 h to allow for appropriate senescence induction. There is a significant induction of senescence in all the chemotherapy‐treated groups. There was no significant senescence induction in the control group. Results are presented as means ± SEM (*n* = 3). The asterisk (****) denotes *p* < 0.0001. Carbo, carboplatin; Dxr, doxorubicin; Gem, Gemcitabine; Pxt, paclitaxel.

**FIGURE 4:**
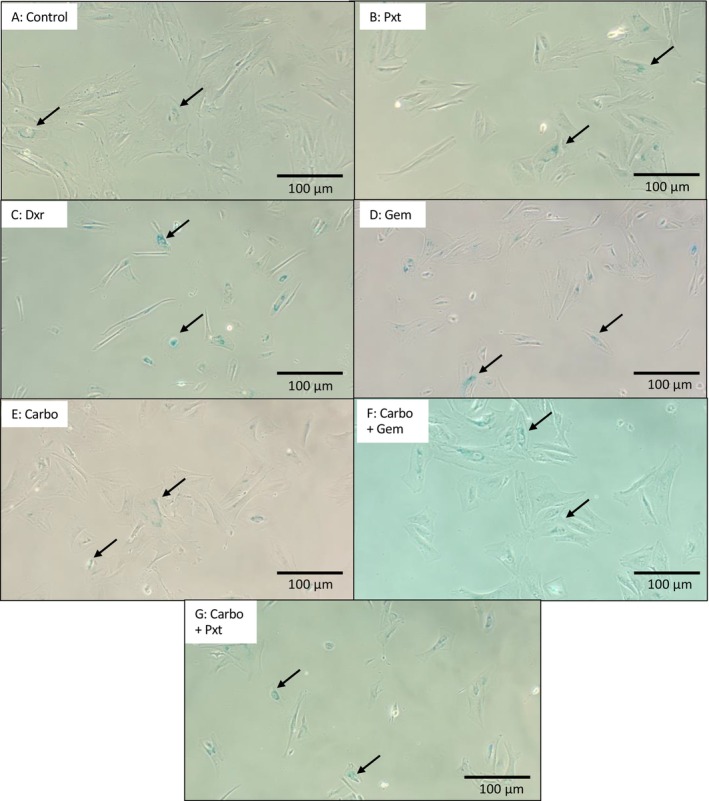
Images of senescence induction via beta‐galactosidase staining. Images were acquired at a 10×X magnification (scale bar of 100 μm) on a Zeiss phase contrast microscope. Carbo, carbo‐platin; Dxr, oxorubicin; Gem, emcitabine; Pxt, aclitaxel.

Senescent cells show increased lysosomal activity, including higher SA‐ß‐gal levels, which thrive at a pH of 6 [11], making SA‐ß‐gal a key marker for senescence. To confirm senescence induction in the treatment groups, SA‐ß‐gal staining was performed (Figure 3). Results are expressed as a percentage out of 100, showing significant senescence induction in all chemotherapy groups compared to the control. Representative images are shown in Figure 4.

SA‐ß‐gal forms when substrates like lactose and sphingolipids are cleaved by the ß‐gal enzyme. This lysosomal hydrolase is visualised by staining with an SA‐ß‐gal kit (Cell Signalling Technology), producing a blue dye. The observed senescence induction across all treatment groups aligns with the WST‐1 results, which showed reduced cell viability. Notably, Pxt had the lowest senescence induction and reduction in cell viability. However, similar senescence levels across treatments highlight the need to analyse specific markers to better understand the underlying mechanisms and pathway activation.

### 3.3 The Effects of Senescence Induction on Cellular Proliferation

**FIGURE 5:**
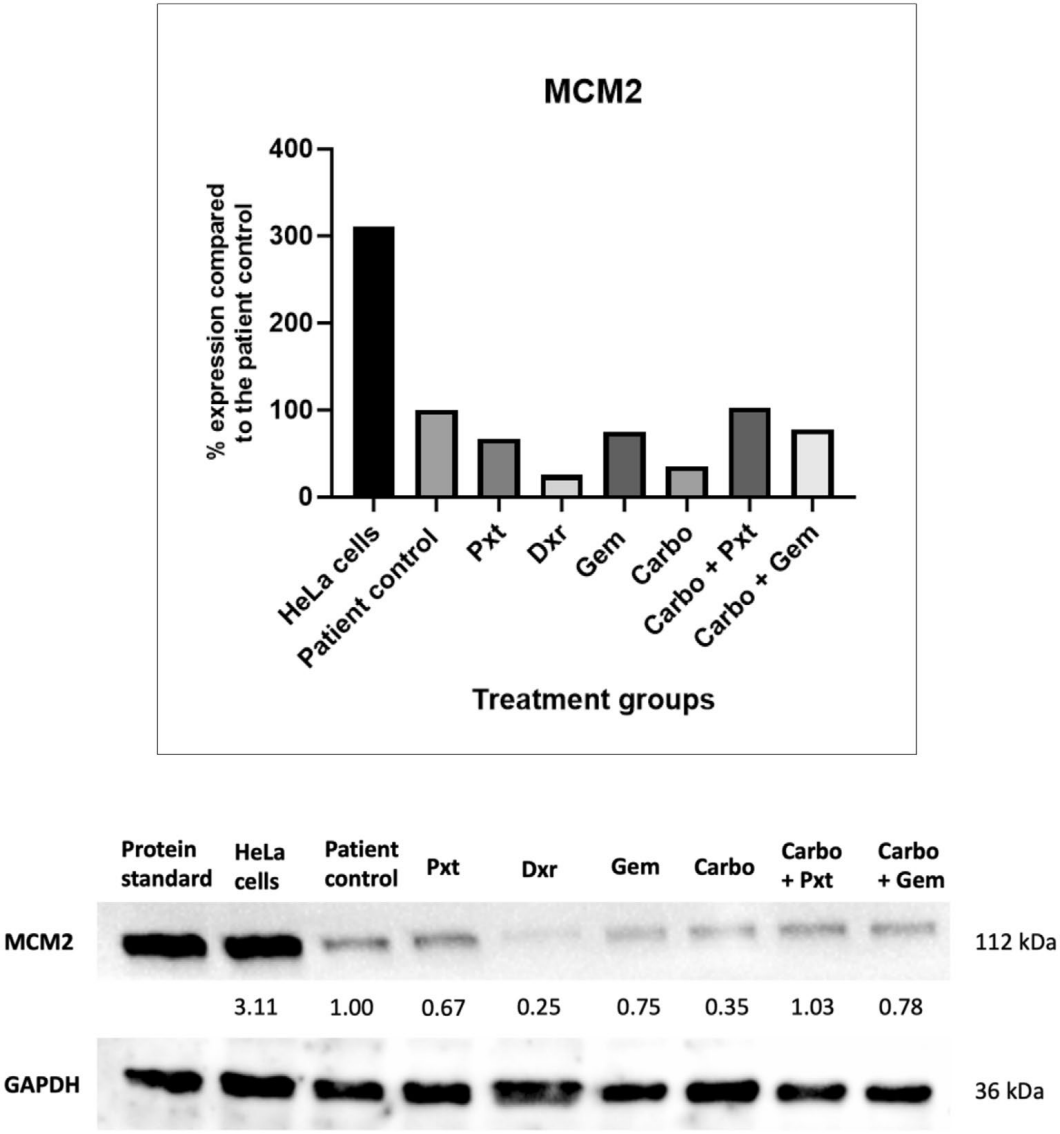
Protein expression of proliferation marker MCM2 via western blot analysis. The patient cells were seeded and treated for 72 h in T25 flasks with the appropriate chemotherapy concentrations. Results are expressed relative to the patient control and normalised to a housekeeping protein, GAPDH. Carbo, carboplatin; Dxr, doxorubicin; GAPDH, glyceraldehyde‐3‐phosphate dehydrogenase; Gem, gemcitabine; kDa, kilodaltons; Pxt, paclitaxel.

Western blot analysis of the proliferation marker MCM2, which regulates DNA replication and cell proliferation [19], was conducted to investigate the observed changes in cell viability and senescence induction. A decrease in MCM2 expression indicates reduced proliferation in the chemotherapy groups compared to the control. Figure 5 shows reduced MCM2 levels in the Pxt, Dxr, Gem, Carbo and Carbo + Gem groups. An immortalised HPV‐positive HeLa cell line served as a positive control.

The Carbo + Pxt group showed increased MCM2 expression, indicating activation of proliferation pathways. In contrast, reduced MCM2 levels were observed in the other treatment groups, aligning with the WST‐1 results (Figure 2) and suggesting decreased proliferation. The Dxr group showed the lowest MCM2 expression, followed by Carbo. Although Carbo is widely preferred for cervical cancer treatment, its cell viability results did not reflect this advantage. Nonetheless, the low MCM2 expression may point to reduced proliferation in the Carbo group.

### 3.4 Confirmation of Senescence Induction Markers

**FIGURE 6:**
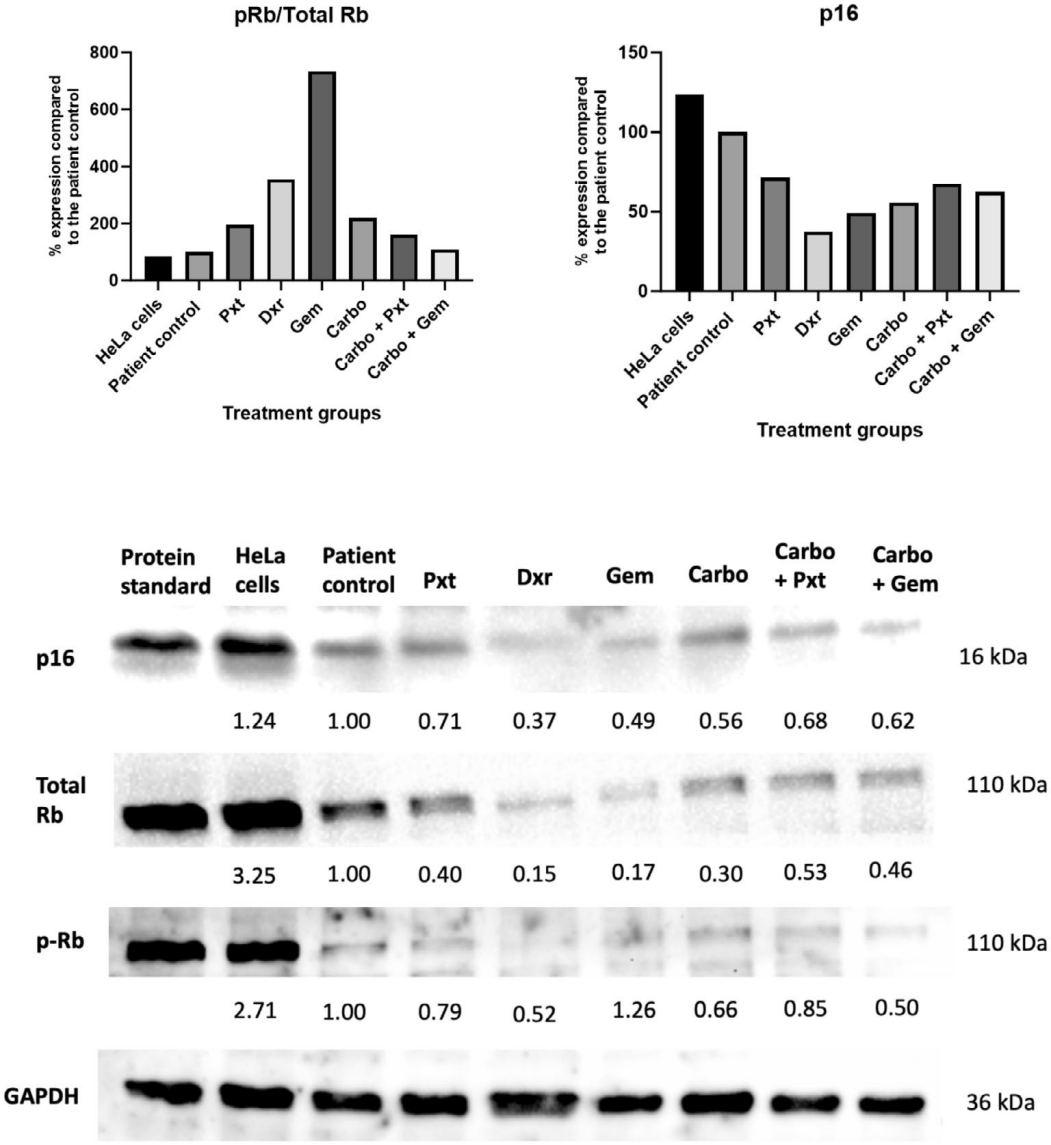
Protein expression of senescence markers p16, Rb and p‐Rb via western blot analysis. The patient cells were seeded and treated for 72 h in T25 flasks with the appropriate chemotherapy concentrations. Results are expressed relative to the control and normalised to a housekeeping protein, GAPDH. Carbo, carboplatin; Dxr, doxorubicin; GAPDH, glyceraldehyde‐3‐phosphate dehydrogenase; Gem, gemcitabine; kDa, kilodaltons; Pxt, paclitaxel.

To further validate senescence induction, western blot analysis examined key markers p16, Rb and p‐Rb (Figure 6). The tumour suppressor protein p16, a regulator of the intrinsic senescence pathway, has strong associations with ageing and senescence [20]. Increased p16 levels indicate heightened senescence through this pathway. Additionally, p16 regulates the p16‐Rb pathway, with Rb playing a crucial role in cell cycle arrest by associating with E2F and promoting hypo‐phosphorylation. The total Rb to p‐Rb ratio serves as an indicator of hypo‐phosphorylation, confirming senescence induction.

All treatment groups showed decreased p16 expression compared to the control, with the greatest reduction in the Dxr group, followed by Gem and Carbo. This reduction suggests decreased p16/Rb pathway activation. The p‐Rb: total Rb ratio aligned with these findings, indicating increased Rb phosphorylation and proliferative pathway activation rather than senescence induction. These results contrast with SA‐ß‐gal staining, which confirmed senescence induction. However, it is possible that an alternative senescence‐induction pathway was activated during this experiment (such as the p21‐p53 pathway and the DNA damage pathway) or that these pathways were not actively engaged at the time of protein harvesting.

Research on chemotherapy and p16 regulation in cervical cancer is limited, though elevated p16 levels have been linked to cisplatin resistance in SiHa cells [21]. The reduced p16 expression, particularly in the Dxr and Gem groups, may suggest a lower likelihood of treatment resistance. Similarly, decreased p‐Rb: Rb expression in these groups further supports this possibility [22]. Interestingly, patients with lower senescence marker levels reportedly achieve better outcomes [23], suggesting the patient in this study might benefit from treatment with Dxr, Gem or Carbo. Nonetheless, further evaluation of treatment resistance markers is essential for drawing definitive conclusions.

### 3.5 Determination of the Presence of Treatment Resistance Markers

**FIGURE 7:**
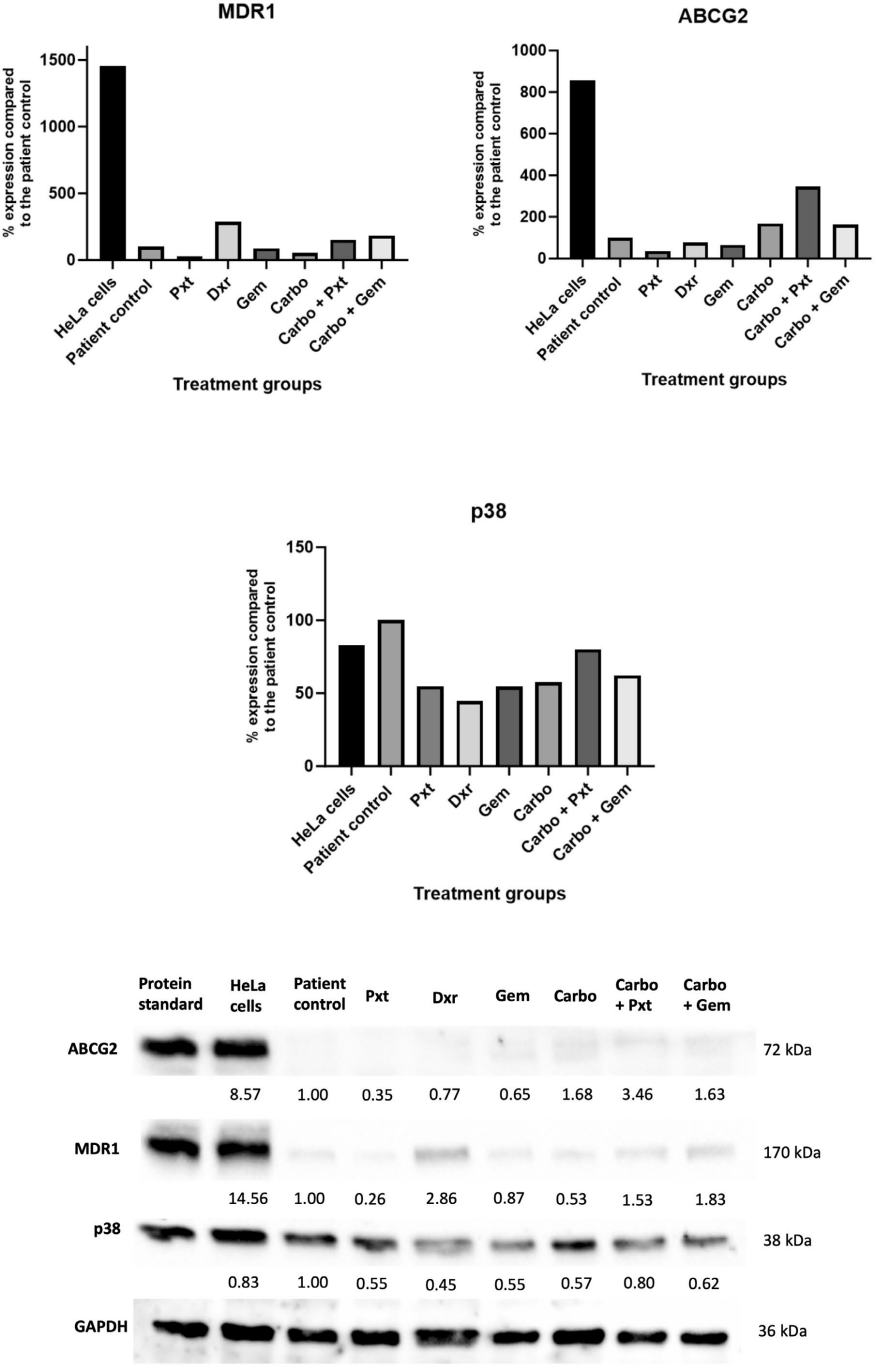
Protein expression of treatment resistance markers ABCG2, MDR1 and p38 via western blot analysis. The patient cells were seeded and treated for 72 h in T25 flasks with the appropriate chemotherapy concentrations. Results are expressed relative to the control and normalised to a housekeeping protein, GAPDH. Carbo, carboplatin; Dxr, doxorubicin; GAPDH, glyceraldehyde‐3‐phosphate dehydrogenase; Gem, gemcitabine; kDa, kilodaltons; Pxt, paclitaxel.

ABCG2 and MDR1 are key markers for assessing treatment resistance, regulated in part by p38 signalling. ABCG2 acts as a multidrug resistance transporter by expelling chemotherapeutics from cancer cells, while MDR1, an ATP‐dependent transporter, plays a similar role in detoxification and resistance [24]. An increase in these markers often indicates drug resistance. Figure 7 shows upregulated ABCG2 in both combination chemotherapy groups, with the Carbo + Pxt group exhibiting the highest expression. MDR1 levels were highest in the Dxr group, followed by the combination groups. Notably, p38 expression decreased across all treatment groups, with the sharpest reductions in the Dxr, Pxt, Gem and Carbo groups.

Chen reported basal ABCG2 expression in cervical cancer tissues, correlating higher levels with advanced disease stages [25]. Despite the patient being at stage 3B, no ABCG2 expression was observed in the control sample. However, treatment with Carbo, Carbo + Pxt and Carbo + Gem led to increased ABCG2 expression, potentially linked to the cancer's advanced stage and a higher likelihood of developing treatment resistance. Notably, heightened ABCG2 expression did not align with the high MCM2 levels observed in combination groups, which typically suggest proliferation. The unexpected ABCG2 increase in the Carbo group, despite low proliferation and senescence marker expression, could be explained by studies showing platinum‐based chemotherapies' role in upregulating ABCG2 in small‐cell lung cancer [26]. Whether this effect extends to cervical cancer remains unclear.

MDR1 overexpression is a known prognostic marker for poor outcomes in cervical cancer, with elevated levels linked to reduced survival rates [27]. In this study, Dxr and the Carbo + Gem combination exhibited the highest MDR1 levels, suggesting a potential association with poorer outcomes. Conversely, Pxt and Carbo alone showed MDR1 levels closest to the control, indicating they may offer better treatment prospects. In a study by Ke et al. Nucleolin, a nucleocytoplasmic protein that is involved in the regulation of MDR1's efflux capabilities, mitigated treatment resistance to cisplatin in HeLa cells in vitro [28]. However, the platinum‐based chemotherapy from the current study, Carbo, did not show the same effects. While cisplatin is associated with greater resistance and severe side effects, Carbo is better tolerated with comparable cytotoxic efficacy [29]. Given the elevated ABCG2 expression with Carbo treatment, its efficacy in mitigating resistance remains uncertain.

Therefore, the relationship between drug resistance and cytotoxicity remains complicated. In this patient, for instance, Dxr treatment resulted in high MDR1 expression and correlating high cytotoxicity, as reflected in the cell viability assays. While this may appear contradictory, since MDR1 is typically associated with drug efflux and reduced efficacy, it likely reflects Dxr's potent ability to induce rapid DNA damage and cell death, despite concurrently triggering cellular stress responses. This stress can upregulate MDR1 and promote cellular senescence. The elevated senescence levels observed in the Dxr group reinforce the idea that while a significant proportion of cells may undergo apoptosis, others may enter a dormant, therapy‐resistant state. These findings highlight that short‐term drug response does not always predict long‐term outcomes, highlighting the importance of evaluating multiple biological endpoints when evaluating treatment outcomes.

### 3.6 Inflammatory Profiling via Blood Plasma Analysis

**FIGURE 8:**
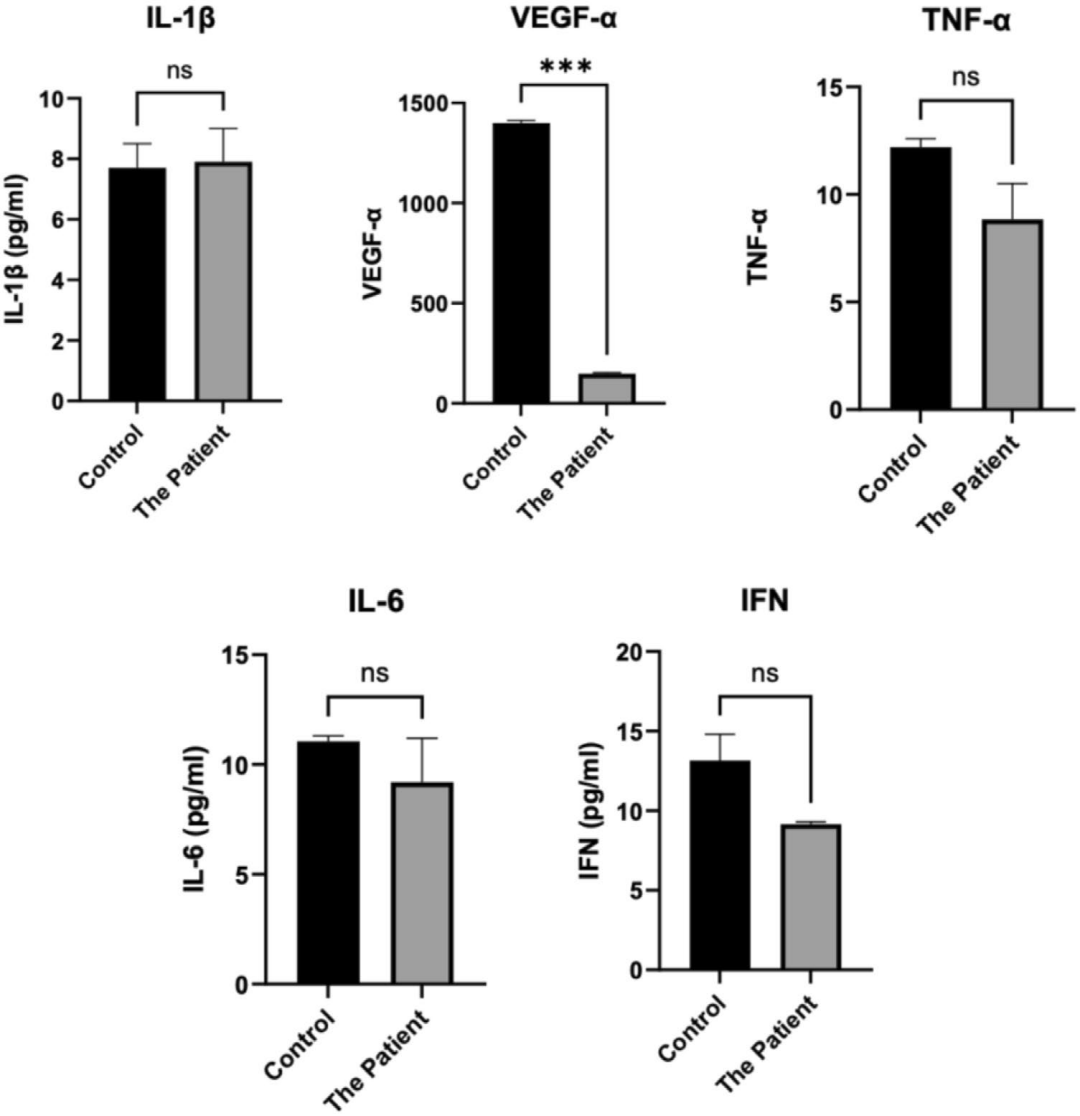
Inflammatory profiling via Milliplex blood plasma analysis. (***p < 0,001).

Inflammatory markers IL‐6, IL‐1ß, TNF‐α, VEGF‐α and IFN were assessed pre‐treatment. These markers are linked to chronic inflammation, contributing to cancer progression, including cervical cancer [30]. Blood analysis (Figure 8) showed no significant differences from the control, except for a notable reduction in VEGF‐α (*p* < 0.001).

Advanced‐stage cervical cancer patients often show elevated levels of IL‐6, IL‐1ß and TNF‐α [30, 31], though this was not observed for the patient. IFN levels also showed no significant reduction, aligning with findings from an Indian cervical cancer cohort [32]. VEGF‐α, typically elevated in solid tumours and linked to metastasis, angiogenesis and proliferation [33], was significantly reduced (*p* < 0.001). Zhang et al. associated high VEGF‐α levels with poor survival outcomes in cervical cancer [34]. Additionally, whole blood plasma analysis was performed (Table 1). While this alone cannot provide definitive prognostic conclusions, it remains vital for guiding chemotherapy decisions and assessing overall health status.

**TABLE 1:** The patient's whole blood plasma analysis.

	White blood cell (WBC) count	Red blood cell (RBC) count	Platelet count
Normal range	3.71 × 10^3^/μL–10.67 × 10^3^/μL	3.87 × 10^6^/μL–5.68 × 10^6^/μL	150.5 × 10^3^/μL–366.8 × 10^3^/μL
Patient results	11.09 × 10^3^/μL	5.21 × 10^6^/μL	231.4 × 10^3^/μL
Indication as ‘high’ or ‘low’ or ‘normal’	High	Normal	Normal

The patient presented with a high white blood cell (WBC) count, while red blood cell (RBC) and platelet counts were normal. Choi et al. identified baseline lymphocyte count as a key prognostic marker for cervical cancer, influencing response to concurrent chemotherapy and radiation [35]. Elevated WBC levels, often linked to inflammation, may impact coagulation and affect chemotherapy outcomes. Although severe WBC elevation can lead to treatment‐related toxicity in advanced cervical cancer [36], this was not the case for the patient. The patient's elevated WBC count may also be attributed to their HIV‐positive status, reflecting the immune response to ongoing infection [37]. These results should be interpreted alongside other findings for a comprehensive assessment.

## 4 The Patient: Conclusions

Considering the results from the WST‐1 cell viability assay, senescence staining, western blot analyses of senescence and treatment resistance markers, as well as blood analysis, determining the optimal treatment strategy for this patient remains challenging. The summarised findings in Table 2 highlight conflicting indicators for an appropriate therapeutic approach.

**TABLE 2:** Summary of the best treatment options for each experimental variable for the patient.

	Cell viability (WST‐1)	Proliferation marker–(MCM2)	Senescence marker–p16	Senescence marker–pRb/total Rb	Treatment resistance marker–MDR1	Treatment resistance marker–ABCG2
Best option	Dxr	Dxr	Dxr	Gem	Pxt	Pxt
Second best option	Carbo + Gem	Carbo	Gem	Dxr	Carbo	Gem

While Dxr demonstrated the most significant reductions in proliferation and cell viability, low‐dosage Carbo emerged as a potentially effective treatment for eliminating cervical cancer cells while avoiding activation of treatment resistance pathways. However, the increased ABCG2 expression associated with Carbo administration raises concerns about its potential to induce treatment resistance. In essence, we hoped that these results could provide valuable insights to clinicians in making well‐informed treatment decisions tailored to the individual patient's needs, rather than relying solely on conventional treatment protocols. Despite the valuable insights gained, experimental limitations and technical challenges necessitated exploring more advanced methodologies, ultimately motivating the investigation of 3D organoid cultures as a means to overcome these restrictions and enhance therapeutic evaluations.

## 5 Organoid Culture

As demonstrated by the results from the patient above and similar findings observed in other patients from this study, definitive conclusions regarding optimal treatment strategies were challenging to draw based solely on ex vivo experiments. To address this limitation, we advanced our approach by incorporating a 3D organoid cell culture model using patient biopsies. Three‐dimensional cancer culturing models are gaining traction due to their enhanced ability to mimic in vivo tumour conditions. Multiple studies have demonstrated that 3D models offer valuable insights into cancer biology and facilitate the identification of cancer cell biomarkers [38, 39, 40]. One significant advantage of 3D culturing lies in its ability to replicate the heterogeneity of drug sensitivity observed in actual tumours. Unlike 2D cultures, which are highly oxygenated and fully exposed to treatment, resulting in rapid drug responses and reactive oxygen species (ROS) accumulation, cells in the hypoxic core of 3D spheroids exhibit greater treatment resistance. These differences in the extracellular matrix (ECM), drug penetration, oxygen and nutrient availability and gene and protein expression profiles make 3D spheroids more accurate representations of the TME. We hypothesised that establishing 3D cultures could offer a more comprehensive approach to evaluating patient‐specific treatment responses and predicting the potential for drug resistance.

### 5.1 Protocol for Organoid Culturing

For organoid culturing, we utilised ClinoStar bioreactors provided by the South African Medical Research Council (SAMRC). These bioreactors facilitate 3D culture by maintaining cells within a continuously rotating vessel filled with culture medium. This dynamic environment promotes spheroid formation while maintaining a stable 3D structure. The ClinoStar system simulates a microgravity‐like environment by applying constant rotation and shear forces, creating a suspension state that encourages uniform cell aggregation and growth. The bioreactor features a central rotating drum and a sealed chamber that precisely controls critical parameters such as oxygen levels, pH and nutrient supply. This setup fosters a stable microenvironment for spheroid development, making it particularly effective for regulating organoid size, shape and physiological conditions. The resulting spheroid cultures are more consistent and reproducible, offering a superior representation of tumour conditions compared to traditional 2D ex vivo models.

In preparation for culturing, biopsy samples were first rinsed with HBSS and then cut into smaller fragments, following a protocol similar to the ex vivo cell culture method. Unlike the ex vivo model, trypsin was not used to dissociate cells into single‐cell suspensions. Instead, intact biopsy fragments were directly distributed across different treatment groups. Ideally, each bioreactor would house 4–6 biopsy pieces, but due to the limited size of the cervical tumour samples, we used 3–5 pieces per bioreactor. After placement in the bioreactors, the samples were incubated in the ClinoStar system until they reached an optimal spheroid diameter of approximately 0.2 mm. At this stage, the organoids were ready for treatment and further experimental analysis.

## 6 Case Report for Patient X

The patient used for the 3D organoid culture, referred to as Patient X, was a 47‐year‐old HIV‐negative South African female diagnosed with stage 2B cervical SCC involving the perimetrium at Tygerberg Hospital, Cape Town. After staging confirmation, she provided informed consent for a cervical tumour biopsy. Due to limited tissue availability, only two treatment groups were used: a control and a carboplatin group, with a 12 μM concentration consistent with ex vivo experiments. Tumour size analysis and IHC staining for the treatment resistance marker ABCG2 were performed.

### 6.1 Protocol for Sectioning and Immunohistochemistry Staining for Patient X Organoids

Patient X's cervical tumour biopsy was cultured in ClinoStar bioreactors for 4 weeks until the organoids reached the experimental size. This lengthy incubation period presented challenges due to time and cost constraints. Moreover, only two viable organoids per bioreactor remained, limiting experimental options. As a result, sectioning and IHC staining were prioritised to maximise analysis.

Organoids were embedded in paraffin wax and sectioned with a microtome. Following deparaffinisation and rehydration, antigen retrieval was conducted using the PASCAL system. ABCG2 primary antibody (1:300 dilution) was incubated overnight at 4°C. After rinsing, HRP‐conjugated secondary antibody was applied for 30 min, followed by DAB chromogen for visualisation. The sections were counterstained with haematoxylin, dehydrated, mounted with coverslips and examined microscopically to assess protein expression.

### 6.2 Analysis of Treatment Resistance Protein Expression Using Organoid Cultures

**FIGURE 9:**
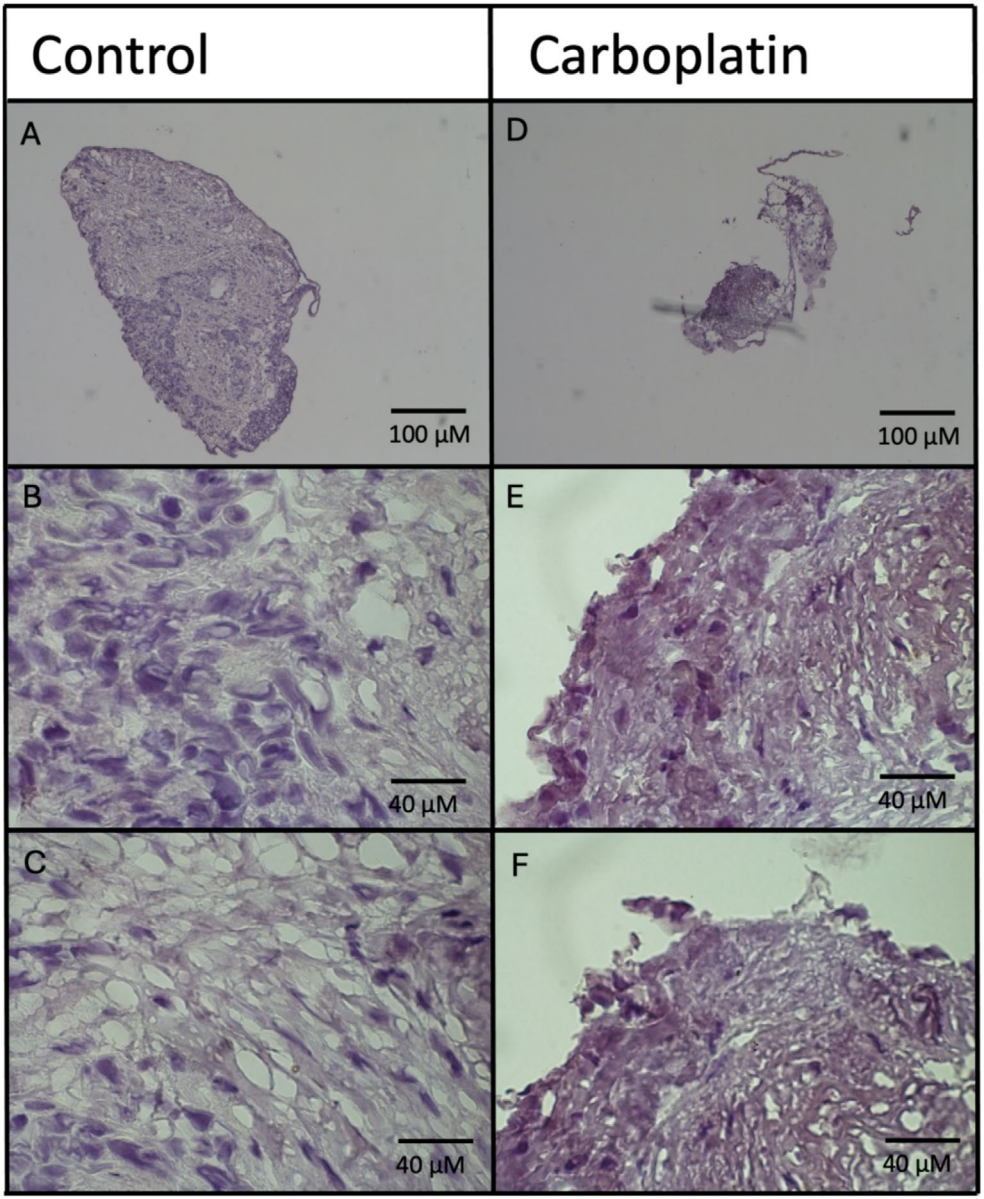
(A–F) IHC staining of treatment resistance marker ABCG2 was done on control and carbo‐treated organoid samples. Images were taken using the Nikon ECLIPSE Ti inverted microscope and NIS elements imaging software at 40× and 100×, respectively.

Analysis of the sample revealed no statistically significant increase in the expression of ABCG2 (*p* < 0.05) compared to the patient control, as shown in Figure 9.

**FIGURE 10:**
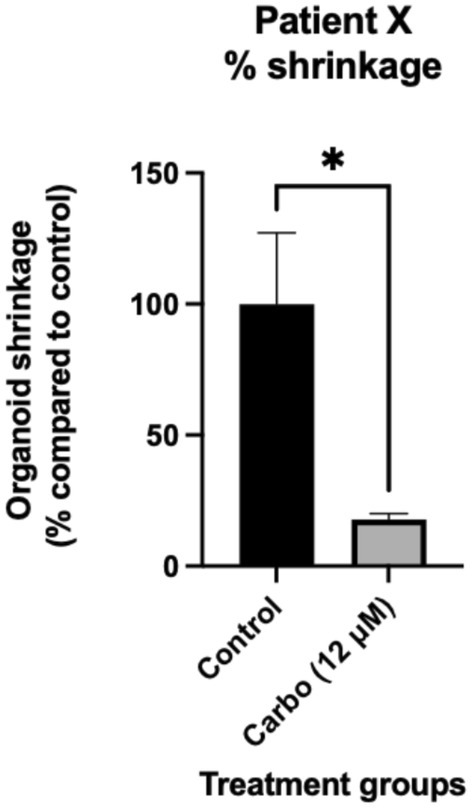
Percentage shrinkage of the organoids after treatment with carbo compared to the control. A significant reduction in the surface area of the organoids was seen in the carbo treatment group compared to the control (*p* < 0.05). Size analysis was done using ImageJ (FIJI) analysis software.

Although no positive ABCG2 staining was observed, carboplatin treatment reduced organoid size by approximately 80% compared to the control (Figure 10).

Using the 3D organoid model aimed to enhance treatment analysis based on ex vivo results. However, culturing challenges, bioreactor limitations and unclear antibody staining highlighted the need for integrative, multidisciplinary approaches in personalised medicine.

## 7 Conclusions

While our initial aim was to predict treatment outcomes for individual patients based on ex vivo cell cultures, the variability and complexity encountered across methods highlighted a broader, critical insight: no single experimental model can fully encompass the nuances of treatment response in cervical cancer. Even though we could not necessarily aid medical practitioners in the best treatment for the patients to reduce the likelihood of drug resistance, these results could be used in combination with other experimental results in the future—once they become optimised and efficient enough—to aid in decision making. These results highlight the importance of expanding experimental optimisation and incorporating multiple methodologies when applying personalised medicine, particularly in resource‐limited regions like South Africa.

While the ex vivo 2D and 3D organoid models demonstrate promising predictive potential, these findings have yet to be correlated with long‐term clinical outcomes, such as progression‐free survival or overall survival. Consequently, future studies are crucial to fully assess the applicability of personalised medicine in LMICs. This need is underscored by the limited availability of molecular diagnostics, high‐throughput sequencing platforms, and biobanking infrastructure in these settings. These constraints highlight the importance of developing simplified, scalable and cost‐effective screening tools that can be feasibly implemented within public healthcare systems. However, even though initial investment costs for incorporating personalised medicine into the public healthcare system may be high, there is growing recognition that such approaches offer considerable long‐term benefits to all key stakeholders by introducing technologies that improve diagnosis, prognosis and treatment; ultimately reducing the number of patients requiring prolonged care and lowering the incidence of adverse drug responses [41].

Additionally, recent advances in organoid technology have demonstrated their potential for modelling tumour architecture and drug response and investigating immune‐tumour interactions [42]. Although the 3D organoid models used in this study do not include immune system components, multiple studies have shown that such integration is feasible and valuable. For instance, co‐culturing patient‐derived organoids with autologous peripheral blood mononuclear cells (PBMCs) has enabled the expansion of T cells capable of targeting matched tumour organoids, effectively modelling immune‐mediated cytotoxicity [43]. These models retain key genetic and histological features of the original tumour, allowing for the assessment of personalised responses to immunotherapies, including immune checkpoint inhibitors. Incorporating immune components such as T cells or tumour‐infiltrating lymphocytes (TILs) into cervical cancer patient‐derived models could enhance their predictive power and relevance for guiding treatment decisions, especially as immunotherapies continue to gain traction in gynaecological oncology. Future work should explore such integrated models to broaden the utility of organoid‐based personalised medicine, particularly in resource‐limited settings where streamlined predictive tools could significantly impact treatment stratification.

This study underscores the value of such an integrative approach to comprehensively understand treatment resistance mechanisms, advocating for carefully designed strategies in personalised medicine research.

## Author Contributions

**Madré Meyer:** investigation, writing – original draft, methodology, writing – review and editing, formal analysis, visualization. **Carla Eksteen:** conceptualization, investigation, validation, methodology, writing – review and editing, project administration, supervision. **Cayleigh de Sousa:** investigation, methodology, data curation. **Nireshni Chellan:** investigation, methodology, resources, data curation. **Ruzayda Van Aarde:** investigation, methodology, data curation, resources. **Meenal Bhaga:** investigation, methodology. **Johann Riedemann:** conceptualization, validation. **Matthys H. Botha:** investigation, resources, methodology. **Frederick H. van der Merwe:** investigation, methodology, resources. **Anna‐Mart Engelbrecht:** conceptualization, funding acquisition, validation, writing – review and editing, project administration, supervision, resources.

## Ethics Statement

Stellenbosch University (HREC—N22/04/037) granted ethical approval for this study.

## Consent

Written informed consent was obtained from the patient to publish this case report and accompanying images. A copy of the written consent is available for review by the Editor‐in‐Chief of this journal on request.

## Conflicts of Interest

The authors declare no conflicts of interest.

## Data Availability

Data sharing not applicable to this article as no datasets were generated or analysed during the current study.
